# O.4.6-10 Move! – A national monitoring system for health-related fitness and motor competence of Finnish school-aged children and adolescents

**DOI:** 10.1093/eurpub/ckad133.229

**Published:** 2023-09-11

**Authors:** Mikko Huhtiniemi

**Affiliations:** University of Jyväskylä, Finland; mikko.huhtiniemi@jyu.fi

## Abstract

**Purpose:**

The Move! system was launched in 2016 and it aims to provide systematic feedback to students on their physical performance, and to collect annual nationwide data on physical performance from 5th and 8th grade students (11–15 years old). The physical performance in the Move! system has been operationalized to include components of health-related fitness and motor competence, which are ideally needed during adolescence and later in life.

**Project description:**

The Move! is included in the national core curriculum for physical education (PE) and is therefore targeted for all students. Move! measurements include 20m shuttle run (endurance), 5-leaps (strength of the lower body, locomotor skills, stability skills), throwing-catching combination (object control skills), curl-up (endurance of abdominal muscles), push-up (endurance of upper body muscles), and flexibility tests. The Move! results are used extensively. In schools, PE teachers give immediate feedback to students during and after test sessions. In addition, teachers can utilize Move! data when designing PE programs and lessons. Students’ results are sent home, and parents are encouraged to discuss the results and their implications with their children. Students and their parents are directed to the Move! web pages, where they are provided with more information about the tests along with guidance on how to develop different elements of physical performance. The results are also utilized in healthcare as school nurses and doctors consider students’ physical performance information along with other health data. Through a database, it is possible to follow students’ physical performance development. The data can also be used in informed decision-making at national and local levels.

**Conclusions:**

The Move! system annually reaches a vast majority of Finnish grade 5 and 8 students (>100 000), schools (>2000), and municipalities (>95 %). The system ties together school PE, health care, and data-informed decision-making, and can be seen as a pedagogical innovation enhancing Finnish students’ health-related fitness, physical activity, and overall well-being.

